# *cis*-Chlorido(ethyl­amine)­bis­(propane-1,3-diamine)­cobalt(III) dichloride

**DOI:** 10.1107/S1600536813004650

**Published:** 2013-02-23

**Authors:** Velusamy Maheshwaran, Viswanathan Thiruselvam, Munisamy Manjunathan, Krishnamoorthy Anbalagan, Mondikalipudur Nanjappa Gounder Ponnuswamy

**Affiliations:** a Centre of Advanced Study in Crystallography and Biophysics, University of Madras, Guindy Campus, Chennai 600 025, India; b Department of Chemistry, Pondicherry University, Pondicherry 605 014, India

## Abstract

In the title compound, [CoCl(C_2_H_7_N)(C_3_H_10_N_2_)_2_]Cl_2_, the Co^III^ ion has a distorted octa­hedral coordination environment and is surrounded by four N atoms in the equatorial plane, with the other N and Cl atoms occupying the axial positions. The crystal packing is stabilized by N—H⋯Cl hydrogen bonds, forming a layered arrangement parallel to (1-10).

## Related literature

For supramolecular structures, see: Desiraju (1995 ▶); Khlobystov *et al.* (2001 ▶); Lehn (1995 ▶); Seo *et al.* (2000 ▶). For Co^III^ complexes, see: Chang *et al.* (2010 ▶). For related and comparable structures, see: Anbalagan *et al.* (2009 ▶); Lee *et al.* (2007 ▶); Ramesh *et al.* (2008 ▶); Ravichandran *et al.* (2009 ▶). For the preparation of (1,3-diamino­propane)­cobalt(III), see: Bailar & Work (1946 ▶).
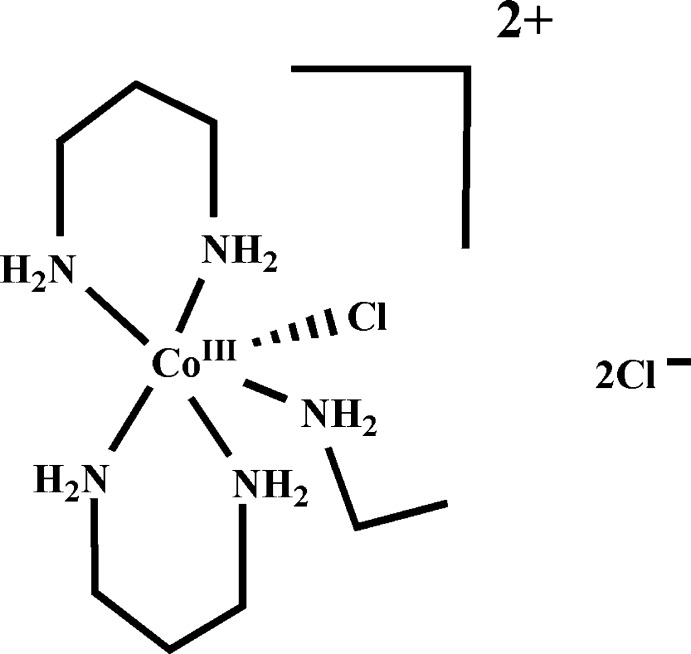


## Experimental

### 

#### Crystal data

[CoCl(C_2_H_7_N)(C_3_H_10_N_2_)_2_]Cl_2_
*M* *_r_* = 358.63
Triclinic, 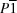

*a* = 7.8847 (4) Å
*b* = 8.0627 (4) Å
*c* = 12.6526 (5) Å
α = 102.780 (3)°
β = 99.936 (4)°
γ = 92.580 (4)°
*V* = 769.76 (6) Å^3^
*Z* = 2
Mo *K*α radiation
μ = 1.62 mm^−1^
*T* = 293 K
0.45 × 0.35 × 0.35 mm

#### Data collection

Oxford Diffraction Xcalibur Eos diffractometer
Absorption correction: multi-scan (*CrysAlis PRO*; Oxford Diffraction, 2009 ▶) *T*_min_ = 0.600, *T*_max_ = 1.000
4965 measured reflections
2711 independent reflections
2299 reflections with *I* > 2σ(*I*)
*R*_int_ = 0.018

#### Refinement

*R*[*F* ^2^ > 2σ(*F* ^2^)] = 0.025
*wR*(*F* ^2^) = 0.060
*S* = 0.99
2711 reflections
194 parameters
H atoms treated by a mixture of independent and constrained refinement
Δρ_max_ = 0.30 e Å^−3^
Δρ_min_ = −0.35 e Å^−3^

### 

Data collection: *CrysAlis CCD* (Oxford Diffraction, 2009 ▶); cell refinement: *CrysAlis RED* (Oxford Diffraction, 2009 ▶); data reduction: *CrysAlis RED*; program(s) used to solve structure: *SHELXS97* (Sheldrick, 2008 ▶); program(s) used to refine structure: *SHELXL97* (Sheldrick, 2008 ▶); molecular graphics: *ORTEP-3 for Windows* (Farrugia, 2012 ▶) and *PLATON* (Spek, 2009 ▶); software used to prepare material for publication: *PLATON*.

## Supplementary Material

Crystal structure: contains datablock(s) global, I. DOI: 10.1107/S1600536813004650/bt6888sup1.cif

Structure factors: contains datablock(s) I. DOI: 10.1107/S1600536813004650/bt6888Isup2.hkl

Additional supplementary materials:  crystallographic information; 3D view; checkCIF report

## Figures and Tables

**Table 1:** Hydrogen-bond geometry (Å, °)

*D*—H⋯*A*	*D*—H	H⋯*A*	*D*⋯*A*	*D*—H⋯*A*
N2—H2*D*⋯Cl2^i^	0.84 (2)	2.77 (2)	3.5033 (19)	145.6 (18)
N3—H3*C*⋯Cl2^i^	0.79 (2)	2.49 (2)	3.275 (2)	168 (2)
N1—H1*D*⋯Cl2^ii^	0.82 (2)	2.52 (2)	3.3278 (19)	173.2 (18)
N3—H3*D*⋯Cl2^ii^	0.87 (2)	2.72 (2)	3.472 (2)	145.6 (18)
N4—H4*C*⋯Cl3^iii^	0.88 (2)	2.40 (2)	3.261 (2)	163.7 (19)
N5—H5*D*⋯Cl3^iii^	0.82 (2)	2.57 (2)	3.329 (2)	154 (2)

Symmetry codes: (i) 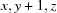; (ii) 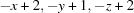; (iii) 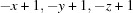.

**Fig. 1.**
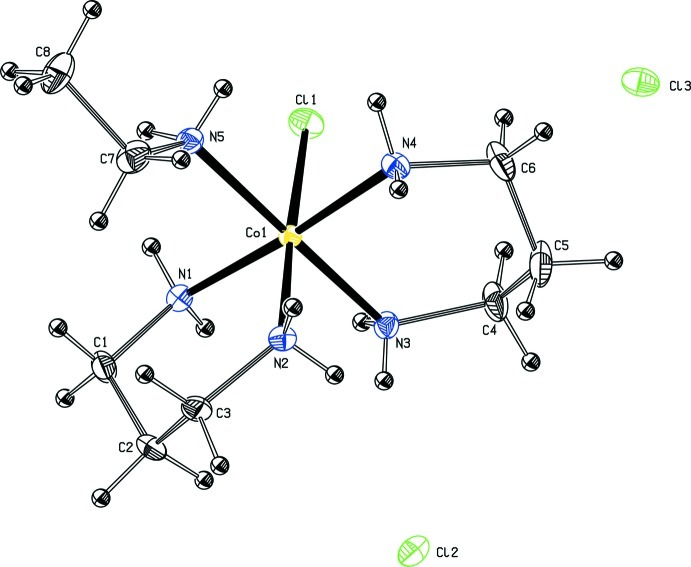
The molecular structure of the title compound, showing the atomic numbering and displacement ellipsoids drawn at 30% probability level.

**Fig. 2.**
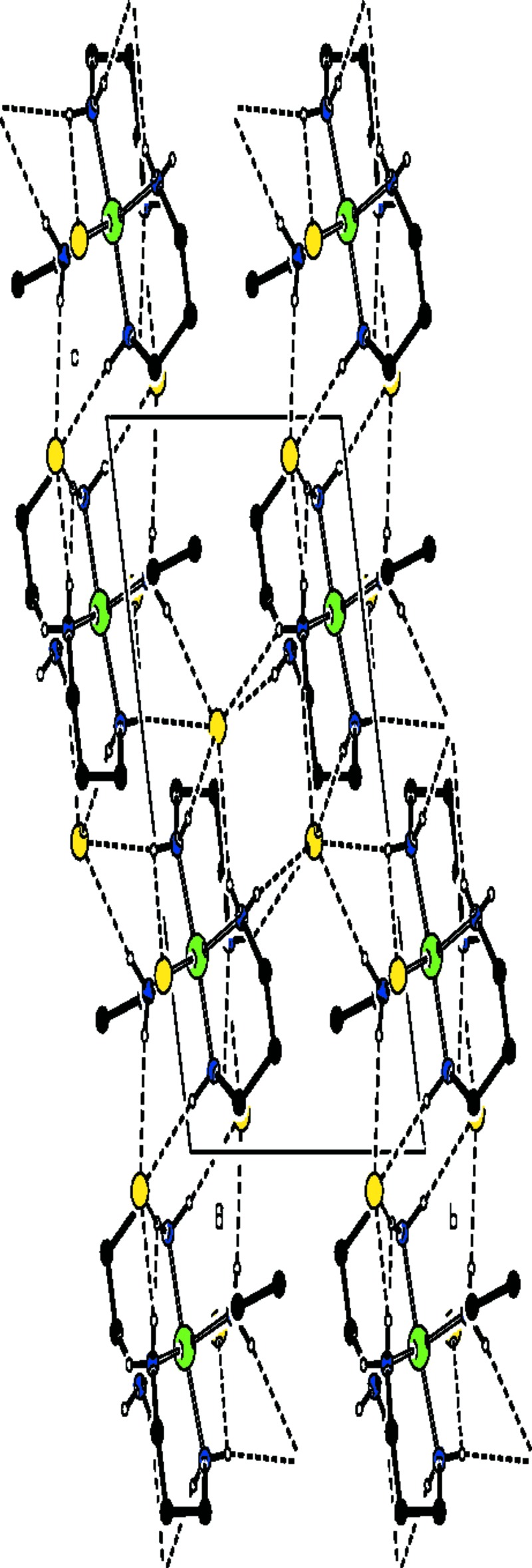
The packing of the molecules viewed down a axis.

## supplementary crystallographic information

### Comment

In recent years, considerable effort has been dedicated to the design and synthesis of supramolecular architectures of coordination complexes (Lehn, 1995; Khlobystov *et al.*,2001). The primary reason for the interest in such complexes is their new and versatile topologies and potential applications in functional materials (Desiraju, 1995; Seo *et al.*, 2000).

The interaction of transition metal polyamine complexes of cobalt(III) with DNA has received considerable attention in the recent years. Using mixed ligand complexes, it is possible to systematically vary parameters of interest by changing the properties of the interacting units either by the use of suitable substituents or simply by changing the nature of ancillary ligand.

In addition, cobalt(III) complexes have received a sustained high level of attention due to their relevance in various redox processes in biological systems and act as promising agents for antitumor, anthelmintic, antiparasitic, antibiotic and antimicrobial activities, as well as their multiple applications in fields of medicine and drug delivery (Chang *et al.*, 2010). Against this background and to ascertain the molecular structure and conformation of the title compound, the crystal structure determination has been carried out.

The *ORTEP* plot of the molecule is shown in Fig. 1. The molecular geometry is not a perfect octahedron. The metal centre is surrounded by four N atoms in an equatorial plane, with the other N and Cl atoms occupying the axial positions.

The bond lengths [Co—N] and [Co—Cl] are comparable with the values reported in the literature (Lee *et al.*, 2007; Ramesh *et al.*, 2008; Anbalagan *et al.*, 2009; Ravichandran *et al.*, 2009).

The packing of the molecules viewed down the *a* axis is shown in Fig. 2. The packing is stabilized by N—H···Cl and N—H···N types of inter- and intramolecular interaction.

### Experimental

2 grams of *trans*-[Co^III^(tn)2Cl_2_]Cl solid was made in the path using 3–4 drops of water. To the solid mass, about 0.12*M* ethyl amine (EtNH_2_) was dropped for 20 min and mixed well. The grinding was continued until the colour turned dull green to red (Bailar & Work, 1946). The reaction mixture was set aside until no further change was observed and the product was allowed to stand overnight. Finally, the solid was washed. The final solid was dissolved in 5–10 ml of water pre-heated to 70°C and allowed to crystallize using hot acidified water. Finally Microcrystalline pink color crystal was retrieved (yield 0.85 g). The crystals were filtered, washed with ethanol and dried over vacuum. X-ray quality crystals were obtained by recrystallization from hot acidified distilled water.

### Refinement

All H atoms were discernable in a difference map. C-bound H atoms were positioned geometrically (C–H =0.93–0.97 Å) and allowed to ride on their parent atoms, with *U*_iso_(H) =1.5*U*_eq_(C) for methyl H atoms and 1.2*U*_eq_(C) for all other H atoms. The H atoms bonded to N were freely refined.

### Crystal data

**Table:**

[CoCl(C_2_H_7_N)(C_3_H_10_N_2_)_2_]Cl_2_	*Z* = 2
*M**_r_* = 358.63	*F*(000) = 376
Triclinic, *P*1	*D*_x_ = 1.547 Mg m^−^^3^
Hall symbol: -P 1	Mo *K*α radiation, λ = 0.71073 Å
*a* = 7.8847 (4) Å	Cell parameters from 2784 reflections
*b* = 8.0627 (4) Å	θ = 3.4–29.0°
*c* = 12.6526 (5) Å	µ = 1.62 mm^−^^1^
α = 102.780 (3)°	*T* = 293 K
β = 99.936 (4)°	Block, yellow
γ = 92.580 (4)°	0.45 × 0.35 × 0.35 mm
*V* = 769.76 (6) Å^3^	

### Data collection

**Table:**

Oxford Diffraction Xcalibur Eos diffractometer	2711 independent reflections
Radiation source: fine-focus sealed tube	2299 reflections with *I* > 2σ(*I*)
Graphite monochromator	*R*_int_ = 0.018
ω scans	θ_max_ = 25.0°, θ_min_ = 2.8°
Absorption correction: multi-scan (*CrysAlis PRO*; Oxford Diffraction, 2009)	*h* = −9→9
*T*_min_ = 0.600, *T*_max_ = 1.000	*k* = −9→9
4965 measured reflections	*l* = −15→12

### Refinement

**Table:**

Refinement on *F*^2^	Primary atom site location: structure-invariant direct methods
Least-squares matrix: full	Secondary atom site location: difference Fourier map
*R*[*F*^2^ > 2σ(*F*^2^)] = 0.025	Hydrogen site location: inferred from neighbouring sites
*wR*(*F*^2^) = 0.060	H atoms treated by a mixture of independent and constrained refinement
*S* = 0.99	*w* = 1/[σ^2^(*F*_o_^2^) + (0.036*P*)^2^] where *P* = (*F*_o_^2^ + 2*F*_c_^2^)/3
2711 reflections	(Δ/σ)_max_ = 0.001
194 parameters	Δρ_max_ = 0.30 e Å^−^^3^
0 restraints	Δρ_min_ = −0.35 e Å^−^^3^

### Special details

**Table:**

Geometry. All e.s.d.'s (except the e.s.d. in the dihedral angle between two l.s. planes) are estimated using the full covariance matrix. The cell e.s.d.'s are taken into account individually in the estimation of e.s.d.'s in distances, angles and torsion angles; correlations between e.s.d.'s in cell parameters are only used when they are defined by crystal symmetry. An approximate (isotropic) treatment of cell e.s.d.'s is used for estimating e.s.d.'s involving l.s. planes.
Refinement. Refinement of *F*^2^ against ALL reflections. The weighted *R*-factor *wR* and goodness of fit *S* are based on *F*^2^, conventional *R*-factors *R* are based on *F*, with *F* set to zero for negative *F*^2^. The threshold expression of *F*^2^ > σ(*F*^2^) is used only for calculating *R*-factors(gt) *etc*. and is not relevant to the choice of reflections for refinement. *R*-factors based on *F*^2^ are statistically about twice as large as those based on *F*, and *R*- factors based on ALL data will be even larger.

### Fractional atomic coordinates and isotropic or equivalent isotropic displacement parameters (Å^2^)

**Table:**

	*x*	*y*	*z*	*U*_iso_*/*U*_eq_	
C1	0.7176 (3)	0.6154 (3)	0.98937 (17)	0.0278 (5)	
H1A	0.7705	0.6146	1.0644	0.033*	
H1B	0.6287	0.5210	0.9632	0.033*	
C2	0.6360 (3)	0.7792 (3)	0.98931 (16)	0.0289 (5)	
H2A	0.7265	0.8717	1.0056	0.035*	
H2B	0.5691	0.8005	1.0477	0.035*	
C3	0.5193 (3)	0.7803 (3)	0.88121 (16)	0.0253 (5)	
H3A	0.4308	0.6858	0.8637	0.030*	
H3B	0.4622	0.8853	0.8896	0.030*	
C4	1.0645 (3)	0.9053 (3)	0.74479 (16)	0.0364 (6)	
H4A	1.1607	0.8388	0.7295	0.044*	
H4B	1.1112	1.0209	0.7820	0.044*	
C5	0.9486 (4)	0.9081 (3)	0.63747 (19)	0.0460 (7)	
H5A	0.8456	0.9630	0.6526	0.055*	
H5B	1.0082	0.9744	0.5975	0.055*	
C6	0.8976 (3)	0.7309 (3)	0.56745 (17)	0.0359 (6)	
H6A	0.8494	0.7373	0.4927	0.043*	
H6B	0.9996	0.6683	0.5650	0.043*	
C7	0.4777 (3)	0.3755 (3)	0.7108 (2)	0.0333 (5)	
H7A	0.4207	0.4316	0.6557	0.040*	
H7B	0.4483	0.4289	0.7811	0.040*	
C8	0.4119 (3)	0.1879 (3)	0.6799 (2)	0.0432 (7)	
H8A	0.2888	0.1776	0.6750	0.065*	
H8B	0.4656	0.1326	0.7353	0.065*	
H8C	0.4396	0.1349	0.6100	0.065*	
N1	0.8505 (2)	0.5908 (2)	0.91895 (14)	0.0205 (4)	
N2	0.6139 (2)	0.7664 (2)	0.78807 (14)	0.0202 (4)	
N3	0.9726 (3)	0.8316 (2)	0.81887 (14)	0.0210 (4)	
N4	0.7690 (3)	0.6375 (3)	0.61050 (14)	0.0223 (4)	
N5	0.6662 (2)	0.3992 (2)	0.71863 (16)	0.0222 (4)	
Cl1	1.03872 (7)	0.47044 (7)	0.74016 (4)	0.03006 (14)	
Cl2	0.79985 (7)	0.17837 (6)	0.92229 (4)	0.02905 (14)	
Cl3	0.36383 (7)	0.73553 (7)	0.55285 (4)	0.03125 (14)	
Co1	0.80944 (3)	0.62246 (3)	0.766006 (19)	0.01506 (9)	
H2C	0.543 (3)	0.741 (3)	0.7294 (17)	0.024 (6)*	
H3C	0.926 (3)	0.908 (3)	0.8502 (17)	0.020 (7)*	
H3D	1.052 (3)	0.797 (3)	0.8632 (17)	0.022 (6)*	
H4D	0.685 (3)	0.678 (3)	0.5978 (17)	0.019 (7)*	
H1C	0.882 (3)	0.490 (3)	0.9103 (16)	0.020 (6)*	
H4C	0.756 (3)	0.532 (3)	0.5693 (16)	0.020 (6)*	
H2D	0.652 (3)	0.867 (3)	0.7894 (17)	0.030 (7)*	
H5D	0.693 (3)	0.360 (3)	0.659 (2)	0.037 (7)*	
H1D	0.937 (3)	0.652 (3)	0.9535 (17)	0.024 (6)*	
H5C	0.717 (3)	0.339 (3)	0.763 (2)	0.042 (8)*	

### Atomic displacement parameters (Å^2^)

**Table:**

	*U*^11^	*U*^22^	*U*^33^	*U*^12^	*U*^13^	*U*^23^
C1	0.0319 (13)	0.0330 (12)	0.0235 (11)	0.0038 (10)	0.0115 (10)	0.0120 (10)
C2	0.0376 (14)	0.0307 (12)	0.0203 (11)	0.0054 (10)	0.0138 (10)	0.0030 (9)
C3	0.0241 (12)	0.0242 (11)	0.0291 (11)	0.0077 (9)	0.0117 (9)	0.0033 (9)
C4	0.0341 (14)	0.0469 (15)	0.0264 (12)	−0.0202 (12)	0.0066 (11)	0.0086 (11)
C5	0.0562 (17)	0.0496 (16)	0.0346 (13)	−0.0224 (14)	0.0048 (12)	0.0233 (12)
C6	0.0319 (13)	0.0576 (16)	0.0186 (11)	−0.0099 (12)	0.0059 (10)	0.0113 (11)
C7	0.0252 (12)	0.0287 (12)	0.0428 (13)	−0.0039 (10)	0.0049 (11)	0.0037 (10)
C8	0.0386 (15)	0.0320 (14)	0.0556 (16)	−0.0137 (11)	0.0004 (13)	0.0130 (12)
N1	0.0189 (10)	0.0188 (10)	0.0230 (9)	0.0011 (8)	0.0022 (8)	0.0047 (8)
N2	0.0198 (10)	0.0212 (10)	0.0184 (9)	0.0030 (8)	0.0005 (8)	0.0039 (8)
N3	0.0210 (10)	0.0211 (10)	0.0206 (9)	−0.0018 (8)	0.0032 (8)	0.0051 (8)
N4	0.0196 (11)	0.0259 (11)	0.0188 (9)	−0.0004 (9)	0.0013 (8)	0.0021 (8)
N5	0.0224 (10)	0.0205 (9)	0.0213 (10)	−0.0017 (8)	0.0027 (8)	0.0016 (8)
Cl1	0.0230 (3)	0.0349 (3)	0.0332 (3)	0.0107 (2)	0.0084 (2)	0.0057 (2)
Cl2	0.0321 (3)	0.0204 (3)	0.0321 (3)	−0.0007 (2)	0.0000 (2)	0.0061 (2)
Cl3	0.0277 (3)	0.0351 (3)	0.0248 (3)	0.0028 (2)	0.0000 (2)	−0.0022 (2)
Co1	0.01382 (15)	0.01541 (15)	0.01497 (15)	0.00020 (10)	0.00225 (10)	0.00205 (10)

### Geometric parameters (Å, º)

**Table:**

C1—N1	1.481 (3)	C7—H7A	0.9700
C1—C2	1.495 (3)	C7—H7B	0.9700
C1—H1A	0.9700	C8—H8A	0.9600
C1—H1B	0.9700	C8—H8B	0.9600
C2—C3	1.513 (3)	C8—H8C	0.9600
C2—H2A	0.9700	N1—Co1	1.9811 (17)
C2—H2B	0.9700	N1—H1C	0.85 (2)
C3—N2	1.486 (2)	N1—H1D	0.82 (2)
C3—H3A	0.9700	N2—Co1	1.9921 (18)
C3—H3B	0.9700	N2—H2C	0.83 (2)
C4—N3	1.483 (2)	N2—H2D	0.84 (2)
C4—C5	1.506 (3)	N3—Co1	1.9887 (17)
C4—H4A	0.9700	N3—H3C	0.79 (2)
C4—H4B	0.9700	N3—H3D	0.87 (2)
C5—C6	1.502 (3)	N4—Co1	1.9698 (18)
C5—H5A	0.9700	N4—H4D	0.76 (2)
C5—H5B	0.9700	N4—H4C	0.88 (2)
C6—N4	1.482 (3)	N5—Co1	1.9953 (16)
C6—H6A	0.9700	N5—H5D	0.82 (2)
C6—H6B	0.9700	N5—H5C	0.88 (3)
C7—N5	1.474 (3)	Cl1—Co1	2.2591 (6)
C7—C8	1.519 (3)		
			
N1—C1—C2	112.37 (17)	H8B—C8—H8C	109.5
N1—C1—H1A	109.1	C1—N1—Co1	122.70 (14)
C2—C1—H1A	109.1	C1—N1—H1C	111.0 (14)
N1—C1—H1B	109.1	Co1—N1—H1C	102.2 (13)
C2—C1—H1B	109.1	C1—N1—H1D	106.7 (15)
H1A—C1—H1B	107.9	Co1—N1—H1D	108.1 (14)
C1—C2—C3	113.66 (17)	H1C—N1—H1D	105 (2)
C1—C2—H2A	108.8	C3—N2—Co1	124.93 (14)
C3—C2—H2A	108.8	C3—N2—H2C	108.5 (15)
C1—C2—H2B	108.8	Co1—N2—H2C	106.5 (15)
C3—C2—H2B	108.8	C3—N2—H2D	106.4 (15)
H2A—C2—H2B	107.7	Co1—N2—H2D	105.9 (16)
N2—C3—C2	112.96 (17)	H2C—N2—H2D	102 (2)
N2—C3—H3A	109.0	C4—N3—Co1	122.95 (13)
C2—C3—H3A	109.0	C4—N3—H3C	105.6 (15)
N2—C3—H3B	109.0	Co1—N3—H3C	109.9 (15)
C2—C3—H3B	109.0	C4—N3—H3D	105.5 (14)
H3A—C3—H3B	107.8	Co1—N3—H3D	100.6 (14)
N3—C4—C5	112.45 (18)	H3C—N3—H3D	112 (2)
N3—C4—H4A	109.1	C6—N4—Co1	120.95 (14)
C5—C4—H4A	109.1	C6—N4—H4D	105.3 (16)
N3—C4—H4B	109.1	Co1—N4—H4D	109.1 (16)
C5—C4—H4B	109.1	C6—N4—H4C	105.5 (13)
H4A—C4—H4B	107.8	Co1—N4—H4C	107.6 (13)
C6—C5—C4	111.3 (2)	H4D—N4—H4C	108 (2)
C6—C5—H5A	109.4	C7—N5—Co1	125.65 (15)
C4—C5—H5A	109.4	C7—N5—H5D	110.9 (17)
C6—C5—H5B	109.4	Co1—N5—H5D	100.9 (16)
C4—C5—H5B	109.4	C7—N5—H5C	109.0 (16)
H5A—C5—H5B	108.0	Co1—N5—H5C	103.4 (16)
N4—C6—C5	111.91 (19)	H5D—N5—H5C	105 (2)
N4—C6—H6A	109.2	N4—Co1—N1	176.20 (8)
C5—C6—H6A	109.2	N4—Co1—N3	94.63 (7)
N4—C6—H6B	109.2	N1—Co1—N3	88.27 (8)
C5—C6—H6B	109.2	N4—Co1—N2	88.57 (8)
H6A—C6—H6B	107.9	N1—Co1—N2	93.94 (7)
N5—C7—C8	111.9 (2)	N3—Co1—N2	89.27 (9)
N5—C7—H7A	109.2	N4—Co1—N5	88.27 (8)
C8—C7—H7A	109.2	N1—Co1—N5	88.62 (8)
N5—C7—H7B	109.2	N3—Co1—N5	173.98 (9)
C8—C7—H7B	109.2	N2—Co1—N5	96.09 (8)
H7A—C7—H7B	107.9	N4—Co1—Cl1	90.49 (7)
C7—C8—H8A	109.5	N1—Co1—Cl1	87.13 (6)
C7—C8—H8B	109.5	N3—Co1—Cl1	88.21 (7)
H8A—C8—H8B	109.5	N2—Co1—Cl1	177.23 (6)
C7—C8—H8C	109.5	N5—Co1—Cl1	86.49 (6)
H8A—C8—H8C	109.5		
			
N1—C1—C2—C3	70.5 (2)	C1—N1—Co1—N5	−75.46 (17)
C1—C2—C3—N2	−64.4 (2)	C1—N1—Co1—Cl1	−162.02 (16)
N3—C4—C5—C6	−68.7 (3)	C4—N3—Co1—N4	−20.0 (2)
C4—C5—C6—N4	73.7 (3)	C4—N3—Co1—N1	157.5 (2)
C2—C1—N1—Co1	−48.3 (2)	C4—N3—Co1—N2	−108.52 (19)
C2—C3—N2—Co1	37.7 (2)	C4—N3—Co1—Cl1	70.34 (19)
C5—C4—N3—Co1	43.3 (3)	C3—N2—Co1—N4	161.39 (17)
C5—C6—N4—Co1	−51.5 (3)	C3—N2—Co1—N1	−15.75 (17)
C8—C7—N5—Co1	−175.91 (15)	C3—N2—Co1—N3	−103.96 (17)
C6—N4—Co1—N3	23.7 (2)	C3—N2—Co1—N5	73.28 (17)
C6—N4—Co1—N2	112.81 (19)	C7—N5—Co1—N4	−89.73 (19)
C6—N4—Co1—N5	−151.1 (2)	C7—N5—Co1—N1	92.47 (19)
C6—N4—Co1—Cl1	−64.58 (19)	C7—N5—Co1—N2	−1.35 (19)
C1—N1—Co1—N3	109.69 (17)	C7—N5—Co1—Cl1	179.67 (18)
C1—N1—Co1—N2	20.54 (17)		

### Hydrogen-bond geometry (Å, º)

**Table:**

*D*—H···*A*	*D*—H	H···*A*	*D*···*A*	*D*—H···*A*
N2—H2*D*···Cl2^i^	0.84 (2)	2.77 (2)	3.5033 (19)	145.6 (18)
N3—H3*C*···Cl2^i^	0.79 (2)	2.49 (2)	3.275 (2)	168 (2)
N1—H1*D*···Cl2^ii^	0.82 (2)	2.52 (2)	3.3278 (19)	173.2 (18)
N3—H3*D*···Cl2^ii^	0.87 (2)	2.72 (2)	3.472 (2)	145.6 (18)
N4—H4*C*···Cl3^iii^	0.88 (2)	2.40 (2)	3.261 (2)	163.7 (19)
N5—H5*D*···Cl3^iii^	0.82 (2)	2.57 (2)	3.329 (2)	154 (2)

Symmetry codes: (i) *x*, *y*+1, *z*; (ii) −*x*+2, −*y*+1, −*z*+2; (iii) −*x*+1, −*y*+1, −*z*+1.
